# Surgery of Stomach

**Published:** 1905-01-07

**Authors:** 


### SURGERY OF STOMACH.

*(Concluded from page 248.)*

**Gastric Dilatation and Tetany.**—Cunningham[10] says tetany dependent upon gastric dilatation and known as gastric tetany is to be differentiated from the tetanic spasms occurring in gastro-enteritis, pregnancy, thyroidectomy, the puerperal state, acute fever, and the carpopedal spasms associated with rickets; also from those produced from toxine substances, as in lead and morphine poisoning etc.; and from the diseases of the nervous system, such as epilepsy, hysteria, and the definite lesions of the spinal cord, *e.g.*, syringomyelia. The attack of tetany most commonly follows severe vomiting, or less frequently after emptying and lavage of the stomach. Usually the first symptom is pricking and numbness in the hands. This early symptom, as a rule, is of so little inconvenience to the patient that it is frequently overlooked, unless the surgeon puts the question direct. Later in the disease there may be an aura of fatigue, and the attack is usually preceded by severe vomiting. Tetanic contractions appear in the hands, the fingers being flexed at the metacarpophalangeal joints, and the outer phalanx usually extended. The thumb is adducted into the palm of the hand. The wrists are either thoroughly flexed or less frequently extended. The lower arm is flexed at the elbow and rotated across the chest. The legs are flexed at the hips and knees, the ankles and toes flexed or extended. The face occasionally shows trismus, with the corners of the mouth considerably drawn out. Transitory blindness has been recorded. The patient may remain conscious and quiet, motor speech being involved, or delirious with absence of sensory speech. The muscles of the neck, chest, back, and abdomen are less commonly involved. The limbs are sometimes tense and œdematous, and the patient may be cyanotic. The attacks last for a few minutes, occasionally or frequently repeated, or may continue for days and weeks. These prolonged cases are almost invariably fatal. As the attack subsides, pressure over the principal nerve trunks or blood-vessels, enough to impede the venous or arterial circulation, will reproduce the paroxysm (Trousseau's symptom). The medical treatment is directed towards emptying the stomach of its residuum and keeping it so, thereby eliminating whatever toxic substances may enter the circulation or cause the tetany from this source, and also to thus improve the tone of the stomach and the motor insufficiency. At the onset of the attack the stomach should be emptied by the stomach tube if possible. Ice over the spine or repeated warm baths have been recommended. Bromides and morphine are to be given during the attack. Between the attacks thorough lavage with antiseptics is to be practised to prevent putrefaction. The cases treated medically have, however, a mortality of about 80 per cent., but treatment by gastro-enterostomy, pyloroplasty, etc., has given a mortality of about 37 per cent. only. Most of the patients treated surgically were *in extremis*, and, considering the improved technique of gastric surgery, it is probable that this mortality would be much diminished by earlier operation. Whatever may be the especial cause or effect upon the general system of the symptom gastric tetany, is speculative; yet it is certainly severe and much more serious than gastric dilatation alone, from whatever cause. The primary underlying factor is pyloric obstruction; the inability of the patient to hold the proper amount of nutrition in the stomach; or, if able to retain the sufficient nutrition, the inability through motor insufficiency to force it into the intestines because of the small lumen of the pylorus. The pyloric obstruction, if sufficient to cause dilatation, motor insufficiency, and stasis, and to produce gastric tetany, is too serious a condition to be overcome by medical treatment, and operative interference—gastro-enterostomy or some equivalent—is necessary to establish a sufficient outlet of the stomach into the intestines. Moynihan[11] observed gastric tetany five times in a hundred cases of gastro-enterostomy. Probably the condition was due to old-standing gastric dilatation, and would have been prevented by an earlier operation.

**Hour-Glass Stomach** may take many forms according to Moynihan.[12] As a rule the constriction is near the middle of the organ, or near the pyloric end. The isthmus may be very much indurated, or it may be tubular with very little or no induration, but with some thickening. In most instances the greater curvature seems to be pulled upwards towards the lesser. The causes of acquired hour-glass stomach are:—1. Perigastric adhesion. 2. Chronic ulcer. 3. Malignant disease. The following are the signs of hour-glass stomach:—1. If the stomach tube be passed and the stomach washed out with a known quantity of fluid, the loss of a certain quantity will be observed when the return fluid is measured. 2. If the stomach be washed out until the fluid returns clear, a sudden rush of foul-smelling fluid may occur; or if the stomach be washed clean, the tube withdrawn and passed again in a few minutes, several ounces of dirty offensive fluid may escape. 3. Paradoxical dilatation. If the stomach be palpated and a succussion splash obtained, the stomach tube passed, and the stomach apparently emptied, palpation will still elicit a distinct splashing sound. 4. Von Eiselsberg observed in one of his cases that on distending the stomach a bulging of the left side of the epigastrium was produced; after a few moments this gradually subsided, and concomitantly there was a gradual filling up and bulging on the right side. 5. Von Eiselsberg also noticed a bubbling, forcing, "sizzling" sound which can be heard when the stethoscope is applied over the stomach after distension with carbonic acid gas formed by the administration of a seidlitz powder in two halves. 6. The stomach resonance is percussed. A seidlitz powder in two halves is then administered. On percussing, after about 20 or 30 seconds, an enormous increase in the resonance of the upper part of the stomach can be found, while the lower part remains unaltered. 7. Schmidt-Monard and Eichhorst have both seen a distinct nucleus between the two pouches distended with carbonic acid gas. 8. When the stomach is filled with water and examined by gastro-diaphany, the trans-illumination is seen only in the cardiac pouch; the pyloric pouch remaining dark. 9. The deflatable rubber bag of Turck and Hemmeter is passed and distended. The bulging caused thereby is limited to the cardiac pouch, which lies to the left of the middle line. Operation is the only means of cure. More than one operation may be required. Thus in a series of cases the following operations were performed: gastro-plasty alone, gastro-enterostomy alone, gastro plasty and gastro-enterostomy, gastro-gastrostomy alone, and gastro-gastrostomy with gastro-enterostomy. Anastomosis from the second pouch is obviously futile. Brook [13] relates a case of congenital hour-glass stomach treated unsuccessfully by gastro-plasty.

[10] Ibid., p. 527. [11] Ibid., p. 695. [12] Brit. Med. Jour., Feb. 20, 1904, p. 413. [13] Ibid., May 7, 1904, p. 1073.

